# Intranasal carvacrol nanoemulsion: Stroke imaging and neuroprotective study

**DOI:** 10.5599/admet.2967

**Published:** 2025-12-02

**Authors:** Hitendra Mahajan, Ghanashyam Girnar

**Affiliations:** Department of Pharmaceutics, R. C. Patel Institute of Pharmaceutical Education and Research, Shirpur, Maharashtra, India

**Keywords:** Nose to brain, radiolabelling, gamma scintigraphy, middle cerebral artery occlusion

## Abstract

**Background and purpose:**

Cerebral ischemia causes neuronal damage due to restricted blood flow and presents significant challenges for brain-targeted drug delivery because of the blood-brain barrier. The purpose of this study is to formulate and evaluate an intranasal oil-in-water nanoemulsion of carvacrol, a neuroprotective agent, for direct nose-to-brain delivery in treating cerebral ischemia.

**Experimental approach:**

Nanoemulsions were developed using high-pressure homogenization and optimized by adjusting surfactant mixture ratios and homogenization cycles. Characterization included droplet size (<200 nm), polydispersity index, zeta potential, drug content, transmission electron microscopy (TEM), and drug release profiles. Intranasal suitability was assessed through *in vitro*, *ex vivo* and *in vivo* studies, including pharmacokinetics, single photon emission computed tomography (SPECT) imaging, and evaluation in a middle cerebral artery occlusion rat model.

**Key results:**

The optimized nanoemulsion showed sustained release, enhanced nasal permeation, and significantly improved brain bioavailability (*C*_max_ = 67.79 ng mL^-1^ intranasal *vs.* 49.71 ng mL^-1^ intravenous). Lower systemic exposure and high targeting indices (DTE: 1317 %; DTP: 92.40 %) confirmed efficient brain delivery. SPECT imaging validated localized uptake, and the ischemia model demonstrated strong neuroprotective efficacy.

**Conclusion:**

Intranasal carvacrol-loaded nanoemulsion is a promising non-invasive strategy for cerebral ischemia treatment, enabling effective brain targeting and significant therapeutic benefit.

## Introduction

Stroke causes lasting disability in about 795,000 people yearly. Ischemic stroke is the most prevalent and life-threatening type, marked by high rates of mortality and recurrence. Narrow time windows limit treatments like thrombolysis and thrombectomy. Rapid neuronal death occurs in infarcted regions following cerebral ischemia (CI) [1,2].

Ischemic stroke treatments are limited. Tissue plasminogen activator offers modest benefits when administered promptly; novel bioactive peptides, such as carnosine, and connexin-based compounds show promise for minimizing brain damage and aiding recovery. However, their clinical effectiveness still needs further validation [3]. Thrombolytic therapy is underused, even in advanced healthcare systems. Delays and irreversible damage limit its success, benefiting only 12-25 % of stroke patients. There is a pressing need for more effective, timely treatments [4].

Natural products show promise against stroke. Bioactive compounds possess antioxidant, anti-apoptotic and anti-inflammatory properties that contribute to the protection and preservation of neuronal cells. They are being studied as potential therapies, given how closely their actions match stroke pathology [5,6].

Carvacrol (CV), or 5-isopropyl-2-methylphenol, has emerged as a promising candidate to address these issues. CV, a phenolic monoterpene in essential oils, is abundant in plants like *Thymus, Satureja, Origanum*, and wild bergamot, with concentrations from 12 to 82 %. Its multifunctional nature makes it a promising neuroprotective candidate for ischemic stroke therapy [7,8]. CV is a powerful natural compound with diverse effects, including antioxidant, neuroprotective, anticancer, antidiabetic, and immunomodulatory. It shows promise for human and animal health, food use, and stroke therapy [9-12]. CV is Generally Recognized as Safe (GRAS) as a food additive because it is approved by the U.S. Food and Drug Administration for human use in the food sector and is widely incorporated into food products and spices due to its distinctive flavour and antimicrobial activity [13,14]. It offers neuroprotective benefits beyond culinary use. CV blocks transient receptor potential member 7 (TRPM7), a key driver of anoxic neuronal death, helping limit brain damage after ischemia. This highlights its potential as a natural neuroprotective agent in stroke care [15].

Neurodegenerative and psychiatric disorders are difficult to treat due to the restrictive blood-brain barrier (BBB). Intranasal (IN) drug delivery presents a non-invasive solution, enabling rapid and safe therapeutic access to the brain [16]. The nose-to-brain (NTB) route achieves this by transporting drugs directly through the olfactory and trigeminal nerves, bypassing the BBB and enhancing treatment efficacy, patient compliance, and healthcare cost-efficiency [17]. Although systemic circulation contributes slightly, direct neuronal pathways play a major role. NTB delivery is especially beneficial for compounds with poor oral bioavailability, often affected by first-pass metabolism. Nanoemulsions (NE), owing to their lipid-based, biocompatible nature, significantly improve brain uptake of lipophilic drugs, positioning NE-based IN delivery as a promising platform for central nervous system (CNS) therapeutics, even though the exact NTB mechanisms remain only partially understood [18,19].

Building on this, the current study developed and evaluated a NE formulation of CV for IN delivery, targeting CI. Through advanced characterization techniques, including drug release profiling, transmission electron microscopy (TEM), single photon emission computed tomography (SPECT) imaging, and pharmacokinetic/pharmacodynamic assessments, alongside neuroprotective evaluation via the middle cerebral artery occlusion (MCAO) model, the research aims to establish a novel, effective strategy for CNS therapeutics.

## Experimental

### Materials

CV was purchased from TCI Chemicals (India) Pvt. Ltd, Sesame oil, Polysorbate 80 (Tween 80), and polyethylene glycol 400 (PEG 400) were kind gift samples from Croda India Company Pvt. Ltd., India. High-performance liquid chromatography (HPLC)-grade acetonitrile was procured from Merck Pvt. Ltd., Mumbai, India. All other chemicals and reagents employed in the study were of analytical grade.

### Preparation of carvacrol nanoemulsion

The NE of CV was prepared using the dry gum method. To prepare the primary emulsion, 1 ml of CV was dissolved in 4 ml of sesame oil, which served as the oil phase. The surfactant mixture (Smix), consisting of Tween 80 and PEG 400, was then added to the oil phase in varying compositions. Following this, water was gradually added up to a final volume of 20 ml, with vigorous mixing using a mortar and pestle to form a stable primary emulsion. For the preparation of the carvacrol nanoemulsion (CNE), a high-energy method was employed using a high-pressure homogenizer (HPH), which reduced droplet size and ensured the stability of the final formulation [20,21]. The primary emulsion was diluted to 200 ml with water and subjected to HPH at 80000 kPa and room temperature. The emulsion was passed through the homogenizer for varying numbers of cycles to achieve the desired droplet size and formulation stability. This high-energy processing technique reduced droplet size, yielding a stable, fine CNE suitable for IN delivery. Varying concentrations with different levels of independent factors are mentioned in Table 1.

**Table 1.** Independent variables with different levels

Levels	Independent factors	
	Tween80 : PEG 400 volume ratio	No of cycles
Low	1:0.25	10
Medium	1:0.50	15
High	1:0.75	20

### Preparation of mucoadhesive nanoemulsion

To enhance brain retention and mucosal adhesion, the prepared CNE of CV was modified by incorporating a 0.5 % aqueous solution of xyloglucan (XG). This mucoadhesive polymer was added dropwise to the CNE, followed by 45 minutes of magnetic stirring to ensure uniform dispersion and stable formulation of the mucoadhesive carvacrol nanoemulsion (MCNE), optimized for IN delivery [22-24].

### Standard calibration curve

Accurately weighed 10 mg of CV was dissolved in 100 mL of ethanol solution to obtain standard solution of 100 μg mL^-1^, and take the aliquots of 0.3, 0.6, 0.9, 1.2, 1.5, 1.8, 2.1 and 2.4 ml of standard solution of 100 μg ml^-1^ and make up the volume up to 10 ml in volumetric flask with ethanol, representing the concentration of 3, 6, 9, 12, 15, 18, 21, 24 μg mL^-1^ and absorbance were taken at *λ*_max_ 275.4 nm by using UV spectrophotometer. A graph of Absorbance vs. concentration was plotted [25].

### Experimental design

Experimental design enables systematic formulation analysis and optimization; notably, central composite rotatable design-response surface methodology (CCRD-RSM) offers superior efficiency and distinct advantages over alternative techniques [26,27]. CCRD-RSM was employed to optimize NE formulation by analysing the effect of surfactant to cosurfactant volume ratios (*X*_1_) and homogenization cycles *(X*_2_), each tested at three levels. Design Expert v7.0.0 guided 13 experimental runs targeting minimal globule size (*Y*_1_*)* and maximal CV content (*Y*_2_) as shown in Table 2. Quadratic models selected via ANOVA (*p* <0.05) confirmed the significant influence of both factors on formulation outcomes.

**Table 2.** Composition of different batches with their results.

No.	Batch code	Tween80 : PEG 400 volume ratio (*X*_1_)	No. of cycles (*X*_2_)	Globule size, nm (*Y*_1_)	Drug content, % (*Y*_2_)
1	C1	1:0.85	15	167.9	28.45
2	C2	1:0.75	10	149.0	12.48
3	C3	1:0.25	20	159.0	10.60
**4**	**C4**	**1:0.50**	**15**	**153.2**	**85.72**
5	C5	1:0.50	15	153.2	85.72
6	C6	1:0.25	10	152.2	27.98
7	C7	1:0.50	15	153.2	85.72
8	C8	1:0.50	22	176.0	48.16
9	C9	1:0.50	8	143.2	21.87
10	C10	1:0.50	15	153.2	85.72
11	C11	1:0.50	15	153.2	85.72
12	C12	1:0.15	15	142.0	12.00
13	C13	1:0.75	20	147.2	34.40

### Characterization of nanoemulsion

NE are thermodynamically unstable systems prone to phase separation due to the hydrophobic effect, in which oil-water phase separation lowers the system's free energy. Physical destabilization may occur via gravitational separation, flocculation, coalescence, Ostwald ripening, or phase inversion. To assess stability, centrifugation studies were performed at 10,000 rpm for 30 minutes using an Optima MAX-XP ultracentrifuge, evaluating phase separation, creaming, and cracking. Heating-cooling stability was examined across four cycles between 4 and 40 °C, with 48-hour storage at each temperature. Formulations that showed no instability were subjected to freeze-thaw stress at -20 to +25 °C for 48 hours per cycle. Those enduring all stress conditions were deemed kinetically stable [28-31].

To assess intrinsic stability, the thermodynamically stable CNE was stored at 4 °C, and phase separation, cracking, and creaming were monitored throughout storage. Kinetic stability was further confirmed by evaluating droplet size at regular intervals [32].

Among all CNE batches, batch C4 demonstrated superior performance and was selected as the optimized formulation for subsequent studies.

The droplet size of optimized CNE and MCNE was measured by dynamic light scattering using a Zetasizer ZS 90, based on intensity fluctuations from Brownian motion. Before analysis, all samples were appropriately diluted with double-distilled water to ensure consistent and accurate measurements [33]. while the zeta potential, indicating surface charge, was assessed by analyzing 0.1 mL of each diluted NE formulation using the same instrument [34].

A small quantity of CNE and MCNE was individually dispersed in ethanol, and 20 μL of the supernatant was drop-cast onto carbon-coated copper grids. Samples were dried under an infrared lamp for 30 minutes and analysed using a JEOL JEM-2100F field-emission TEM (200 kV, 0.1 nm resolution, 50× to 1,500,000× magnification) [35,36].

This viscosity test uses the Brookfield RST Rheometer with spindle CCT40 to assess the flow characteristics of CNE and MCNE. Samples are temperature-adjusted and tested at 500 rpm for 60 seconds across six points to ensure consistent shear conditions and accurate profiling. The multi-point measurement helps identify homogeneity, flow behaviour, and any shear-dependent changes in viscosity. Proper spindle selection, temperature control, and calibration are critical to reliable results [30].

This study was conducted using goat nasal mucosa obtained from a local slaughterhouse. The measurement was performed with a texture analyser (Brookfield Pvt Ltd). The maximal force required for the detachment of samples from the nasal mucosa was used to compute mucoadhesive characteristics of formulations [37].

The refractive index of CNE and MCNE was determined using an Abbe-type refractometer. The samples were analysed in triplicate [25]. Apparent pH of the CNE and MCNE was measured by a pH meter (Systronic 362 μ pH system, India) in triplicate at 25 °C [25].

The osmolarity (*O* / mOsm L^-1^) of the formulations was determined by Equation (1) [38]:



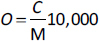

(1)


where *C /* g L^-1^ is concentration of CV *and M* / g is molecular weight of CV.

### In vitro assessment of carvacrol release from nanoemulsions

A dialysis membrane (Himedia, India; molecular weight cutoff: 12,000 to 14,000 kDa) was employed as the diffusion barrier. Membrane segments were pre-soaked in phosphate-buffered saline (PBS, pH 6.4) for 24 hours before placement into Franz diffusion cell assemblies. PBS (pH 6.4; 37 ± 0.5 °C) was added to the receiver compartment, with a constant agitation of 50 rpm. CNE, equivalent to 5 mg of CV, was loaded into the donor chamber. Samples were collected from the receiver chamber at designated time points over 4 hours, with PBS replenishment after each withdrawal. Collected samples were filtered and analysed for CV release using UV spectrophotometry at 275.4 nm [24,39].

For comparison, a plain drug suspension (PDS) containing 5 mg of CV was subjected to identical in vitro release conditions. The same procedure was followed to evaluate CV release from MCNE.

### Ex vivo mucosal permeation investigation

CV release was evaluated through an *ex vivo* diffusion study utilizing Franz diffusion cells and sheep nasal mucosa from CNE, MCNE, and PDS (5 mg dose) as per Raj *et al.* [40] and Mahajan *et al.* [41], with analysis at 275.4 nm.

The steady-state flux *(J*_ss_), apparent permeability coefficient (*P*_app_ / %), and diffusion coefficient (*D*) were derived using Equations (2) to (4), respectively, for both CNE and MCNE [42].



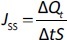

(2)




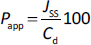

(3)




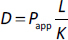

(4)


The term Δ*Q_t_/*Δ*t* shows how fast the drug passes through the nasal surface *Q_t_* / μg cm^-2^ over time, *t* / s. *C*_d_ / mg is the starting amount of CV in the donor side and *S* / cm^2^ is the actual surface area of nasal tissue that the drug contacts. *K* is the partition coefficient, and *L* is the diffusion path length.

### Histological examination

Histological analysis of sheep nasal mucosa was conducted on four sample groups: (A) phosphate buffer solution (PBS) (pH 6.4, negative control), (B) isopropyl alcohol (positive control), (C) CNE and (D)MCNE, following the method described by Picone *et al.* [43] and Keshari *et al.* [44]. MCNE has been selected for further investigation due to its promising results in previous studies.

### Gamma scintigraphy study

Radiolabelling of MCNE was performed using technetium pertechnetate (^99m^Tc) via a reductive method employing stannous chloride (SnCl_2_; 1 mg mL^-1^ in 0.1 N HCl) as the reducing agent. Solution pH was monitored and adjusted as needed, followed by filtration through a 0.22 μm Millipore membrane. A radioactivity dose of 2 mCi of ^99m^Tc was added dropwise, and the mixture was allowed to incubate at ambient temperature for 30 minutes. Radiolabelling efficiency was assessed using ascending instant thin-layer chromatography on silica gel-coated fiberglass sheets (Gelman Sciences, Ann Arbor, Michigan, USA) with the mobile phase 100 % acetone [45-47]. Three formulations containing stannous chloride at 50, 100 and 150 μL were prepared; the formulation with the highest labelling efficiency was selected for further study.

Swiss albino mice received IN administration of radiolabelled ^99m^Tc-MCNE (1 mg kg^-1^). Prior to dosing, animals were partially anesthetized with 4 % isoflurane in oxygen in an induction chamber. A total of 20 μL of MCNE was delivered into each nostril via micropipette, with the mice held in a slanted position to facilitate nasal uptake. Imaging was performed using a bimodal SPECT/CT system (Triumph, Trifoil Imaging Inc., Chatsworth, CA, USA), armed with a gamma camera. After positioning the animals on the imaging bed, SPECT scans were acquired at predetermined time points of 0, 0.5, 1.5 and 3 hours with a 0 to 360° gantry rotation [47-49].

### Pharmacokinetic and brain distribution studies

Male albino Wistar rats (200 to 250 g) were selected for pharmacokinetic and cerebral distribution studies. Animals were housed under standard laboratory conditions: temperature 22±1 °C, relative humidity 55±10 %, and a 12-hour light/dark cycle. During the one-week acclimatization period, rats were provided ad libitum access to a standard pellet diet and potable water. All experimental protocols for pharmacokinetic, pharmacodynamic, and gamma scintigraphy studies were approved by the Institutional Animal Ethics Committee (IAEC/SIPS/2024-25/01B) and conducted by CPCSEA guidelines for the care and use of laboratory animals.

This study explores the pharmacokinetic profile and cerebral distribution of CV following intravenous (IV) and IN administration of MCNE, with a focus on CNS drug delivery efficiency. The study design includes two groups with six animals in each group (*n* = 6) shown in Table 3.

**Table 3.** Different groups for the pharmacokinetic study

Group No	Treatment schedule	Dose
Group 1	MCNE *via* the IN route	1 mg kg^-1^ (3 drops) *per* day
Group 2	MCNE *via* the IV route	1 mg kg^-1^ *per* day

Rats were anesthetized with an intraperitoneal injection of pentobarbital (40 mg kg^-1^) and maintained at 37 °C using a heating pad. Group 1 received 50 μL of the MCNE formulation (1 mg kg^-1^ CV) intranasally using a micropipette, with animals held in a slanted position for optimal delivery. Group 2 administered the same dose intravenously via tail vein injection. Blood samples were collected at designated time intervals via retro-orbital plexus puncture into EDTA-containing tubes, centrifuged for plasma separation, and stored at -20 °C until pharmacokinetic analysis.

Following completion of the protocol, animals were humanely sacrificed, and whole brains were excised. Tissues were rinsed with ice-cold normal saline to remove residual blood and debris, then blotted dry with sterile gauze. Brain samples were weighed and homogenized in methanol (3 mL per 0.3 g tissue) using a Remi homogenizer. The resulting homogenate was centrifuged at 4 °C for 10 minutes at 10,000 rpm. The supernatant was collected, filtered through a 0.45 μm membrane, and stored at -70 °C for subsequent HPLC analysis [50,51].

### Sample analysis

A primary stock solution of CV was prepared by accurately weighing 10 mg and diluting to 100 mL with HPLC-grade methanol. From this, 1 mL was further diluted with 50 mL of methanol to obtain a working concentration of 2 μg mL^-1^. This solution was used to prepare standard solutions at five different concentrations: 10, 50, 100, 150 and 200 ng mL^-1^. Each standard was injected into the HPLC system under optimized chromatographic conditions to establish linearity by plotting peak area against corresponding concentration.

Chromatographic conditions were as follows: C18 column (250×4.6 mm, 5 μm particle size), column temperature 25 °C, mobile phase consisting of acetonitrile and water (70:30 volume ratio), flow rate 1.0 mL min^-1^, UV detection at 270 nm, and an injection volume of 20 μL.

### Statistical data analysis

All data were expressed as mean ± standard deviation (SD). Intergroup differences were assessed using Student’s *t*-test, with statistical significance set at *p* <0.05. Concentration values were dose- and weight-normalized. Non-compartmental analysis using Kinetica 5.0 software was employed to estimate the pharmacokinetic parameters of MCNE. *C*_max_ and *T*_max_ were derived from concentration-time curves for both IN and IV administration routes, while the area under the curve (AUC)_ο-t_ was computed using the trapezoidal rule [52].

### Brain distribution studies

Brain targeting after nasal administration was evaluated using two indices, which can be calculated from Equation (5) and Equation (6) [53,54].

1. Direct targeting efficiency (DTE, %):



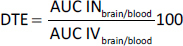

(5)


where AUC IN_brain/blood_ and AUC IN_brain/blood_ represent the brain-to-blood concentration ratios of MCNE following IN and IV administration, respectively.

2. NTB direct transport (DTP, %):



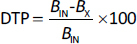

(6)


where, *B*_x_= (*B*_IV_/*P*_IV._)*P*_IN_ is the AUC of brain fraction contributed by systemic circulation through the BBB after IN administration; *B*_IV_ is the AUC_0-240_^*^ for CV measured in brain tissue after IV administration; *P*_IV_ is AUC_0-240_ for CV measured in plasma after IV administration; *B*_IN_ is AUC_0-240_ for CV measured in brain tissue when given IN and *P*_IN_ is AUC_0-240_ for CV measured in plasma after IN administration. DTP elucidates the extent of direct NTB transport.

### Pharmacodynamic study

Pharmacodynamic evaluation of CNE and MCNE was conducted using the MCAO model to induce focal CI in adult male Sprague Dawley rats. Forty-two animals were allocated into six experimental groups (*n* = 7) and maintained under a controlled environment: ambient temperature of 22±2 °C, 12-hour light/dark cycle, and relative humidity of 55±10 %. All rats were group-housed in sterile polypropylene cages and provided ad libitum access to standard chow and filtered water [55]. Male rats weighing 400 to 500 g and aged 8-12 weeks were selected to minimize hormonal variability during neurobehavioral testing [56]. The MCAO model was established *via* the intraluminal filament technique, as described by Longa *et al.* [57] and treatments were administered according to the schedule outlined in Table 4. After 24 hours of reperfusion, rats were euthanized with a high dose of ketamine (100 mg kg^-1^) and xylazine (10 mg kg^-1^) intraperitoneally. The brains were immediately excised, rinsed with PBS, and placed on graph paper for tissue imaging and approximate size estimation. Samples were preserved in PBS for subsequent RP-HPLC for brain homogenate analysis and histological analyses. This method reliably induces transient focal CI, replicating experimental stroke conditions [58]. To account for mortality, two animals were added to each group.

**Table 4.** Treatment schedule for all groups.

Group	Description	Treatment
Group 1	Control	No treatment
Group 2	Sham + Placebo	Placebo (vehicle only)
Group 3	MCAO + CNE (IV)	Dose equivalent to 1 mg kg^-1^ of CV
Group 4	MCAO + CNE (IN)	
Group 5	MCAO + MCNE (IV)	
Group 6	MCAO + MCNE (IN)	

### Infarct volume analysis

Brain images from all six experimental groups were analysed using Image J software. The RGB (Red Green Blue) colour quantification plugin was employed to identify infarcted regions, with red coloration indicating infarction. Quantified red area values were extracted and used to generate a comparative bar graph for visual assessment of infarct severity across groups [59].

### Histopathological examination

Histological analysis of Wistar rat brains was conducted to evaluate MCAO-induced neuronal damage and the neuroprotective effects of CNE and MCNE, following the method prescribed by Pandey *et al.* [19].

### Stability study

To evaluate the physical stability of the CNE and MCNE, the formulation was stored under controlled conditions for a period of six months. Samples were collected at predetermined intervals, specifically at 1, 2, 3 and 6 months and subjected to visual inspection for any signs of sedimentation or phase separation. In addition, each sample was analysed for changes in globule size, polydispersity index (PDI), zeta potential and drug content to assess the integrity and consistency of the formulation over time [51].

## Results and discussion

### Formulation of nanoemulsion

Sesame oil was selected as the oil phase for the NE due to its antioxidant properties, which help improve formulation stability and complement CV. Tween 80 was chosen as the surfactant for its good miscibility with PEG 400, the co-surfactant.

### Standard calibration curve

**Figure 1.**
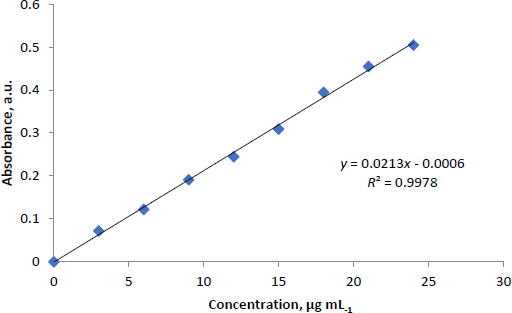
Standard calibration curve of CV

A standard calibration curve was constructed and found to be linear over the range of 0 to 24 μg mL^-1^, indicating compliance with Beer-Lambert’s law; the result is shown in Figure 1.

Limit of detection (LOD) and limit of quantification (LOQ) were calculated from the above data, which were found to be 0.084 and 0.0255 μg mL^-1^, respectively.

### Experimental design

Thirteen CV-loaded NE formulations, with globule sizes ranging from 142-176 nm and drug content between 10.60 to 85.72 %, were analysed using Design Expert® software [33], as shown in Table 2. Quadratic models were applied to both response variables. However, ANOVA indicated that the model for drug content (*Y*_2_) was highly significant and predictive, whereas the globule size (*Y*_1_) model was not significant and showed poor predictive capacity, as summarized in Table 5.

**Table 5.** Regression and ANOVA results, quadratic model

Response	*R^2^*	Adj. *R^2^*	Pred *R^2^*	SD	CV	*p*-value	SS	DF	MS	*F*-value	Significance
*Y*_1_	0.4176	0.0017	3.1411	9.31	6.04	0.4795	434.70	5	86.94	1.00	Not significant
							606.18	7	86.60	-	
							1040.88	12	-	-	
*Y*_2_	0.9913	0.9851	0.9382	4.03	7.89	< 0.0001	12995.68	5	2599.14	159.76	Significant
							113.89	7	16.27	-	
							13109.56	12	-	-	

*R*^2^: coefficient of regression; SD: standard deviation; CV: coefficient of variation, SS: sum of squares; DF: degrees of freedom; MS: mean sum of squares, *F*-value: Fischer's ratio.

**Figure 2.**
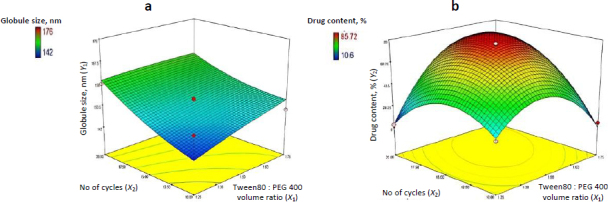
(a) Response surface methodology plot for effect of Smix and number of cycles on (a) globule size and (b) drug content

Independent variables *X*_1_ and *X*_2_ exhibited an adverse effect on globule size, *i.e.* increasing either parameter reduced globule size (*Y*_1_) up to an optimal concentration, as supported by Equation (7) but beyond certain limit it shows opposite effect so the model is suggested as insignificant, this effect can be visualized in the 3D response surface plot (Figure 2a). Both *X*_1_ and *X*_2_ initially showed a positive influence on drug content; however, beyond the optimum levels, further increases led to a decline in drug content (*Y*_2_), as confirmed by Equation (8) and illustrated in Figure 2b.



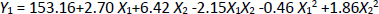

(7)




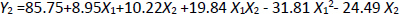

(8)


### Characterization of nanoemulsion

All prepared oil-in-water (o/w) CNE batches underwent thermodynamic stability assessments, including centrifugation, heating-cooling cycles, and freeze-thaw cycles. Among these, batch C4 demonstrated superior stability, showing no signs of phase separation, turbidity, creaming, or cracking. These results suggest that C4 may offer enhanced shelf life and robustness for nanoemulsion formulations.

Batch C4 exhibited kinetic stability over a 90-day period at 4 °C, showing no significant change in globule size (153.2 to 162 nm) or PDI (0.134 to 0.199), indicating minimal aggregation or phase alteration during storage.

**Figure 3.**
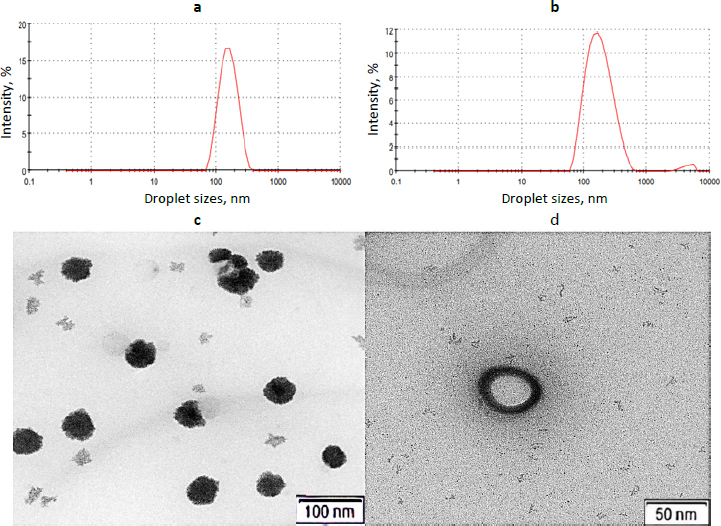
Sizing and TEM visualization of globule (a): globule size of CNE; (b) globule size of MCNE; (c) TEM image of CNE; (d) TEM image of MCNE

Photon correlation spectroscopy confirmed that both CNE and MCNE formulations had droplet sizes below 200 nm (153.2 nm and 162.6 nm, respectively) and low polydispersity indices (0.134 and 0.199), indicating uniform size distributions ideal for IN brain delivery (Figures 3a and 3b, respectively). Zeta potential measurements (−21.0 mV for CNE and -8.16 mV for MCNE) further supported colloidal stability, with CNE's higher negative value suggesting enhanced electrostatic repulsion and potential long-term stability. Overall, both formulations demonstrated favourable physicochemical attributes for targeted CNS delivery.

TEM confirmed nanoscale uniformity across both CNE and MCNE formulations. CNE exhibited compact droplets (~100 nm), while MCNE presented structured spheres (50 to 150 nm) with a layered architecture, indicating high surface area and optimal dispersion key for IN mucosal absorption and brain targeting. Smooth surfaces and small droplet size support consistent drug release, and the absence of aggregation aligns with zeta potential results, reinforcing colloidal stability. These micromorphological features underscore the promise of CNE and MCNE as effective CNS-targeted NE systems (Figure 3c and 3d).

**Figure 4.**
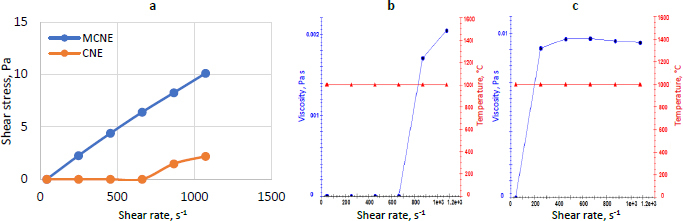
(a) Shear rate *vs.* shear stress for CNE and MCNE, (b) Shear rate *vs.* viscosity for CNE, (c) Shear rate *vs.* viscosity for MCNE

Viscosity and rheological analysis revealed distinct flow behaviours for both NE formulations. Figure 4(a) compares CNE and MCNE by plotting shear stress against shear rate. In Figure 4 (b), the CNE, with a viscosity of 2.0 ± 1.05 mPa·s, exhibited pseudoplastic (shear-thinning) behaviour, where viscosity decreased with increasing shear rate. Rheological modelling using the Herschel-Bulkley equation confirmed its non-Newtonian nature, with a weak yield stress (*τ*_0_ = 0.85 Pa), low consistency index (*K* = 0.6 mPa·s^*n*^), and a flow behaviour index (*n* = 1.45), supporting ease of application and structural responsiveness. In contrast, as shown in Figure 4 (c), the MCNE displayed Newtonian behaviour, maintaining a consistent viscosity of 9.4 ± 2.1 mPa·s across shear rates, with linear shear stress-shear rate correlation and no yield stress. Its average viscosity (*K* = 9.4 mPa·s*^n^*, *n* = 1.0) ensures predictable flow, making it suitable for controlled formulation and processing [60].

**Figure 5.**
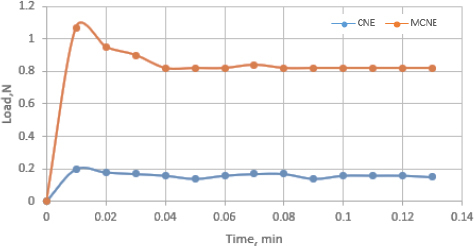
Mucoadhesive study of CNE and MCNE

The force required to detach from the nasal mucosa for MCNE was far better than that for CNE. Figure 5 shows a peak load of 0.20 N for detachment in the case of CNE, which lacks adhesion to the nasal mucosa and a peak load of 1.07 N for detachment for MCNE, attributed to adhesion with the nasal mucosa.

The increased work of adhesion is attributed to the successful coating of NE with XG, which possesses a “mucin-like” molecular structure that confers mucoadhesive properties. This also contributes to improved retention of the formulation in the nasal cavity, thereby achieving better drug absorption.

The refractive index measurements revealed values of 1.34±0.10 for CNE and 1.35±0.10 for MCNE (mean ± SD, *n* = 3). These were compared to the refractive index of water, recorded at 1.33±0.27 (mean ± SD, *n*=3). The comparison showed no statistically significant differences among the three samples. These findings indicate that both CNE and MCNE formulations exhibit optical transparency, aligning closely with the refractive properties of water.

The CNE exhibited a pH of 5.44, while the MCNE showed a pH of 5.20. Both values fall within the acceptable pH range of 4.5 to 6.5 recommended for nasal administration, which is crucial for minimizing the risk of mucosal irritation and ensuring patient comfort.

While the nasal mucosa can tolerate a broad spectrum of tonicity without causing pain, maintaining isotonicity remains crucial for comfort and safety. Nasal formulations with osmolarity levels of 0.5 to 2.0 % sodium chloride (approximately 85.47 to 341.88 mOsmol L^-1^) are generally well tolerated and do not impair nasal ciliary function. The measured osmolarity values for CNE and MCNE were 235.10 and 237.80 mOsmol L^-1^, respectively, well within the recommended range, indicating that these preparations are unlikely to cause irritation or discomfort when administered intranasally [38].

### In vitro assessment of carvacrol release from nanoemulsions

**Figure 6.**
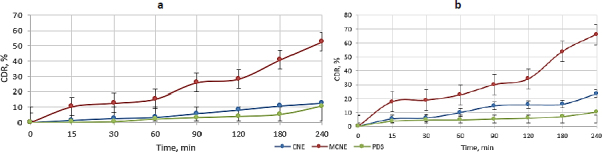
Comparative CDR of CNE, MCNE with PDS (a) *in vitro*; (b) *ex vivo*

Cumulative drug release (CDR, %) profiling revealed significant variations among formulations. The PDS, hindered by poor water solubility, exhibited low and erratic release. In contrast, CNE showed steady release reaching 12.68±0.78 % within 4 hours, while MCNE achieved a substantially higher release of 52.68±1.45 %, indicating superior solubility and enhanced drug release. These results underscore MCNE’s potential for improved therapeutic efficacy over conventional formulations, as shown in Figure 6(a).

### Ex vivo mucosal permeation investigation

As shown in Figure 6 (b), CDR through the recently excised nasal membrane was markedly enhanced by nanoformulations. PDS exhibited limited release (10.30±1.29 %) due to poor solubility, whereas CNE achieved 23.50±0.92 % over 4 hours. MCNE demonstrated the highest release of 66.09±1.44 %, further supporting its enhanced bioavailability, likely due to its mucoadhesive properties.

The permeability study revealed that the MCNE formulation significantly outperformed the CNE in terms of nasal mucosal permeability, suggesting superior penetration. Specifically, MCNE achieved a steady-state flux of 36 ± 2 ng·cm^-2^·s^-1^, markedly higher than CNE’s 5.6 ± 0.12 ng·cm^-2^·s^-1^. The apparent permeability coefficient was 0.360 ± 0.002 μm·s^-1^ for MCNE, compared to 0.129 ± 0.001 μm·s^-1^ for CNE. Similarly, the diffusion coefficient for MCNE (0.0153 ± 0.0020 mm^2^·s^-1^) was nearly triple that of CNE (0.00543 ± 0.0003 mm^2^·s^-1^). These findings indicate that MCNE’s enhanced mucoadhesive characteristics not only improve residence time and reduce mucociliary clearance but also significantly facilitate drug absorption, solidifying its promise as an effective IN delivery system for targeted brain therapies [61].

### Histological examination

**Figure 7.**
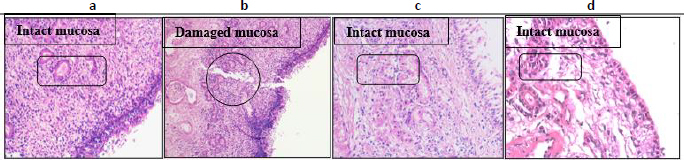
Histological analysis of nasal mucosa post-treatment of (a) PBS pH 6.4; (b) isopropyl alcohol; (c) CNE; (d) MCNE

Histological analysis (Figure 7) showed that nasal tissues treated with CNE and MCNE maintained intact epithelial structures with no signs of inflammation or damage after 2 hours. Compared to control samples, both formulations demonstrated excellent biocompatibility, confirming their safety for IN delivery.

In the above studies, MCNE outperformed CNE, so further research focused solely on MCNE.

### Gamma scintigraphy study

Radiolabelling efficiency of MCNE was evaluated using varying concentrations of stannous chloride (50, 100 and 150 μL). Among these, the 100 μL concentration yielded the highest labelling efficiency at 96.36 % and was selected for subsequent gamma scintigraphy studies.

**Figure 8.**
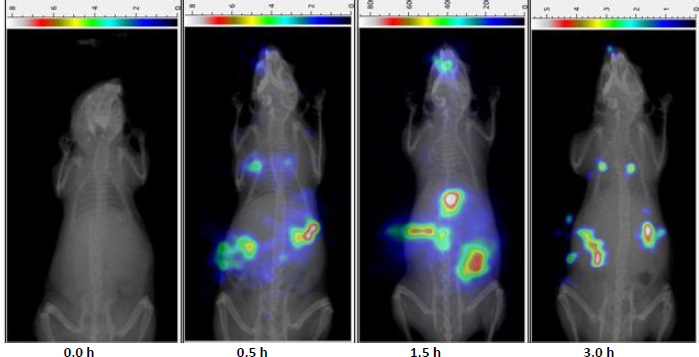
Bimodal SPECT-CT Images indicating brain distribution of MCNE following intranasal administration (0-30 h)

Gamma scintigraphy (Figure 8) indicates successful translocation of the ^99m^Tc-labelled MCNE containing CV to the CNS following IN administration in mice. The observed radioactivity uptake in brain regions suggests that the formulation efficiently reached the CNS via olfactory and trigeminal pathways, bypassing the BBB. These findings align with the outcomes described by Sharma *et al.* [62]. The mucoadhesive nature of MCNE appears to enhance residence time at the nasal mucosa, facilitating targeted and sustained CNS drug delivery.

### Pharmacokinetic study

A validated HPLC method was used to analyse brain homogenate and plasma samples. The calibration curve demonstrated good linearity with an *R*^2^ value of 0.9994, suggesting that Beer-Lambert's law is obeyed. LOD and LOQ values were found to be 6.23 and 18.89 ng mL^-1^, respectively.

*In vivo* pharmacokinetic and brain distribution studies were conducted in male Albino Wistar rats (200 to 250 g) following administration of the MCNE via IV and IN routes. Pharmacokinetic evaluation was performed using a non-compartmental analysis approach.

Comparison of MCNE via IV and IN routes revealed that IN delivery achieved superior brain targeting, with higher concentrations, faster onset, longer retention, and lower systemic exposure. These results highlight IN administration as a promising non-invasive strategy for targeted CV delivery to the brain.

### Plasma pharmacokinetics

**Figure 9.**
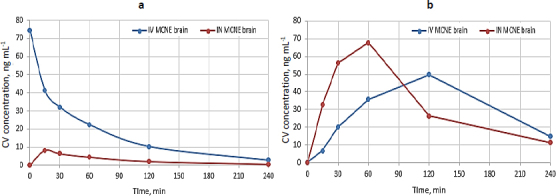
Comparative concentration of CV from MCNE *via* IN and IV routes for (a) plasma and (b) brain.

IV administration showed approximately 9 times higher plasma *C*_max_ (74.44 ng mL^-1^) than IN (8.28 ng mL^-1^), indicating greater systemic exposure, as shown in Figure 9 (a). AUC_0-240_ was 67.26 (IV) *vs.* 11.26 (IN) ng·h mL^-1^, confirming reduced availability *via* the IN route ideal for CNS targeting. *T*_max_ = 0.25 h was delayed for IN, while *T*_½_ = 1.06 h, *K*_el_ = 0.65 h^-1^ and MRT (1.22 *vs.* 1.29 h) were comparable, indicating faster systemic clearance with IN delivery.

### Brain pharmacokinetics

IN administration of MCNE produced a higher brain *C*_max_ (67.79 ng·mL^-1^) compared to IV administration (49.71 ng·mL^-1^) as shown in Figure 9 (b). The brain exposure, expressed as brain AUC_0-240_, was also greater following IN delivery (131.92 ng·h·mL^-1^) than IV (125.81 ng·h·mL^-1^). IN administration achieved a shorter *T*_max_ (1 h) relative to IV (2 h). The elimination half-life(*T*_½_) was longer with IN (1.65 h) than IV (1.14 h), while the elimination rate constant (*K*_el_) was lower (0.42 h^-1^ *vs.* 0.60 h^-1^). Furthermore, the brain AUC was higher for IN (46.99 ng·h·mL^-1^) compared to IV (42.69 ng·h·mL^-1^). Collectively, these findings indicate that IN administration enhances brain uptake and retention of MCNE relative to IV administration. A detailed summary of plasma and brain pharmacokinetic parameters is provided in Table 6.

**Table 6.** Pharmacokinetic parameters

Compartment	Pharmacokinetic parameters	MCNE IV	MCNE IN
Plasma	*C*_max_ / ng mL^-1^	74.44±1.03	8.28±0.50
	*T*_max_ / h	00±0.00	0.25±0.01
	*T*_½_ / h	1.06±0.01	1.06±0.01
	MRT / h	1.29±0.02	1.22±0.03
	*K*_el_ / h^-1^	0.65±0.01	0.65±0.02
	AUC_0-240_ / ng h mL^-1^	67.26±0.83	11.26±0.16
	Peak AUC / ng h mL^-1^	14.98±0.05	1.035±0.02
Brain	*C*_max_ / ng mL-^1^	49.71±0.98	67.79±1.01
	*T*_max_ / h	2±0.02	1±0.01
	*T*_½_ / h	1.14±0.01	1.65±0.02
	MRT / h	1.91±0.02	1.47±0.03
	*K*_el_ / h^-1^	0.60±0.01	0.42±0.03
	AUC_0-240_ / ng h mL^-1^	125.81±0.50	131.92±0.60
	Peak AUC / ng h mL^-1^	42.69±0.28	46.99±0.32

Data expressed as mean ± SD (*n* = 3); *K*_el_: elimination rate constant, MRT: mean residence time

### Brain distribution studies

The exceptionally high DTE of 1317 % and DTP of 92.40 % observed in this study highlight the strong brain-targeting efficiency of the IN MCNE formulation for delivering CV. These findings confirm that a substantial portion of the drug reaches the brain through direct NTB routes, primarily via the olfactory and trigeminal nerves, effectively bypassing systemic circulation. This enhanced delivery aligns with previous reports, which emphasize the importance of small globule size in NE for facilitating transcellular transport across olfactory neurons. In addition to transcellular mechanisms, the extracellular perineural pathway plays a complementary role in NTB transport. This route involves the movement of drug molecules through the perineural spaces surrounding the olfactory and trigeminal nerves, allowing extracellular diffusion into the subarachnoid space and, eventually, into the brain parenchyma. The perineural pathway supports passive, non-cellular transport and is particularly relevant for hydrophilic or larger molecules that may not readily cross neuronal membranes. Furthermore, the high mucoadhesive properties of the formulation contribute to prolonged nasal mucosal residence time, facilitating sustained drug absorption [63-65]. Higher DTE and DTP values confirm superior brain targeting via the IN route [66].

### Pharmacodynamic studies

**Figure 10.**
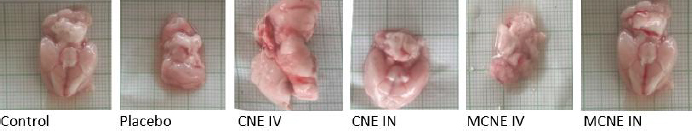
Morphological analysis after MCAO

Morphological analysis post-MCAO (Figure 10) revealed varying degrees of ischemic damage. While the control group showed intact brain structures, the placebo group exhibited severe infarction and oedema. CNE IV group offered partial protection, and CNE IN group preserved architecture more effectively. The MCNEIV group led to inconsistent results, with both damage and localized protection observed. Strikingly, the MCNEIN group showed minimal morphological disruption and well-preserved brain structures, underscoring its promise for neuroprotection in stroke therapy. Mortality was found to be 19.05 %.

### Brain homogenate and infarct volume analysis

**Figure 11.**
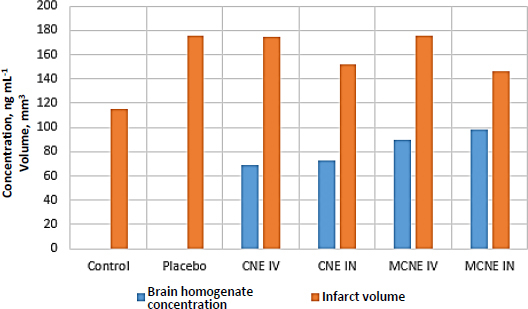
Brain homogenate HPLC analysis and infarct volume analysis after MCAO for the different study groups

Brain images from all six experimental groups were analysed using ImageJ software. The RGB colour quantifycation plugin was employed to identify infarcted regions, with red coloration indicating infarction. Quantified red area values were extracted and used to generate a comparative bar graph for visual assessment of infarct severity across groups. Analysis of infarct volume and CV brain concentrations post-MCAO highlights the superior neuroprotective and brain-targeting efficacy of MCNE administered via the IN route (Figure 11).

The MCNEIN group showed the lowest infarct volume (146.175 mm^3^) and the highest CV concentration in brain tissue (98 ng mL^-1^), outperforming both CNE IN and IV groups. IN-delivered CNE also demonstrated enhanced protection and drug delivery (152.019 mm^3^, 89.48 ng mL^-1^), whereas IV-administered formulations yielded minimal neuroprotection and lower brain CV levels, comparable to placebo. Control animals remained unaffected, serving as baseline references. These results reinforce the therapeutic advantage of MCNE via IN delivery for CI and support its potential in targeted CNS treatment.

### Histopathological evaluation of brain tissue post-MCAO

**Figure 12.**
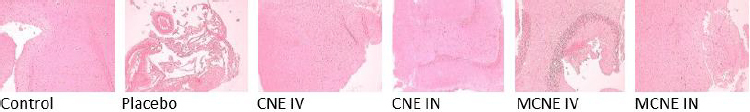
Histopathological analysis of brain tissues from different groups following MCAO

Histological analysis (Figure 12) following MCAO revealed treatment-dependent variations in brain protection. The control group exhibited normal cortical architecture, indicating no pathological changes. In contrast, the placebo group showed severe ischemic injury characterized by neuronal degeneration, gliosis, and pronounced inflammation. Treatment with CNE via the IV route resulted in moderate neuroprotection with reduced inflammation, whereas IN administration of CNE provided better outcomes, including improved tissue integrity and minimal vacuolation. MCNE administered IV led to localized necrosis with moderate preservation of brain tissue, but the most remarkable neuroprotective effect was observed with MCNE IN treatment. This group demonstrated preserved neuronal structure, minimal tissue damage, and reduced inflammation. These findings strongly support the conclusion that MCNE via the IN route offers the highest efficacy in protecting brain architecture following ischemic insult.

### Stability study

Over the six-month study period, both the CNE and MCNE formulations demonstrated promising stability profiles across key parameters shown in Table 7. Drug content remained consistently high in both systems, indicating effective retention of the active ingredient. Globule size increased gradually, as expected over time, yet remained within an acceptable nanoscale range for both formulations. The PDI values showed good uniformity, with both systems maintaining low, stable readings. Zeta potential values, while slightly decreasing, stayed within ranges that suggest reasonable electrostatic stability. Overall, both CNE and MCNE exhibited favourable characteristics, supporting their potential for long-term application in NE-based drug delivery.

**Table 7.** Long-term stability data

Parameter	Time, months									
	0		1		2		3		6	
	CNE	MCNE	CNE	MCNE	CNE	MCNE	CNE	MCNE	CNE	MCNE
Drug content, %	85.72	84.13	85.50	84.01	85.50	83.78	85.20	83.60	84.70	83.50
Globule size, nm	153.2	162.6	159.6	165.9	163.4	168.4	164.8	169.1	167.9	176.6
PDI	0.134	0.199	0.145	0.149	0.179	0.089	0.179	0.153	0.098	0.085
Zeta potential, mV	-21.0	-8.16	-22.1	-8.15	-21.5	-8.18	-21.6	-7.96	-17.2	-6.55

Collectively, these findings highlight the ability of the IN route to bypass the BBB and enable targeted, efficient delivery of neuroprotective agents such as CV.

## Conclusion

The MCNEIN formulation of CV demonstrated remarkable potential for targeted brain delivery, making it highly effective for treating stroke-induced ischemia. Its nanoscale properties, uniform droplet size, high surface area, and stable morphology ensured superior drug release and nasal permeability compared to conventional formulations. Structural integrity was preserved, as confirmed by histology and TEM analyses. Pharmacokinetic studies revealed lower plasma exposure but enhanced brain uptake, showing higher maximum concentration (67.79 ng mL^-1^), prolonged half-life, and greater overall drug retention in the brain. Biodistribution data and gamma scintigraphy confirmed direct NTB transport via olfactory and trigeminal pathways, effectively bypassing the BBB. The formulation achieved an exceptionally high DTE 1317 % and DTP 92.40 %, underscoring its CNS specificity. In stroke models, MCNE IN produced the most significant neuroprotective effects, including reduced infarct volume and preserved neuronal morphology. Overall, this non-invasive approach offers a promising, patient-friendly alternative to conventional routes, delivering rapid, targeted, and sustained brain exposure, ideal for managing acute neurological disorders like ischemic stroke.

## Footnotes

*Concentration of CV in brain when administered by IV route, calculated by AUC from time 0 to 240 minutes.
